# Levels of Evidence for Radiation Therapy Recommendations in the National Comprehensive Cancer Network (NCCN) Clinical Guidelines

**DOI:** 10.1016/j.adro.2021.100832

**Published:** 2021-10-29

**Authors:** Miguel Angel Noy, Benjamin J. Rich, Ricardo Llorente, Deukwoo Kwon, Matthew Abramowitz, Brandon Mahal, Eric A. Mellon, Nicholas G. Zaorsky, Alan Dal Pra

**Affiliations:** a Florida International University, Miami, Florida; b Department of Radiation Oncology, Sylvester Comprehensive Cancer Center, University of Miami Miller School of Medicine, Miami, Florida; c Division of Biostatistics, Department of Public Health Sciences, Sylvester Biostatistics and Bioinformatics Shared Resource, University of Miami Leonard M. Miller School of Medicine, Miami, Florida; d Department of Radiation Oncology, Penn State Cancer Center, Hershey, Pennsylvania; e Department of Public Health Sciences, Penn State College of Medicine, Hershey, Pennsylvania

## Abstract

**Purpose:**

The National Comprehensive Cancer Network (NCCN) clinical guidelines influence medical practice, payor coverage, and standards of care. The levels of evidence underlying radiation therapy recommendations in NCCN have not been systematically explored. Herein, we aim to systematically investigate the NCCN recommendations pertaining to the categories of consensus and evidence (CE) for radiation therapy.

**Methods and Materials:**

We evaluated the distribution of CE underlying current treatment recommendations for the 20 most prevalent cancers in the United States with at least 10 radiation therapy recommendations in the NCCN clinical guidelines. For context, the distribution of evidence in the radiation therapy guidelines was compared with that of systemic therapy using a χ^2^ test. The proportion of category I CE between radiation and systemic therapy was compared using a 2-proportion, 2-tailed z-test in total and for each disease site. A *P* value of < .05 was considered significant.

**Results:**

Among all radiation therapy recommendations, the proportions of category I, IIA, IIB, and III CE were 9.7%, 80.6%, 8.4%, and 1.3%, respectively. When analyzed by disease site, cervix and breast cancer had the highest portion of category I CE (33% and 31%, respectively). There was no radiation therapy category I CE for hepatobiliary, bone, pancreatic, melanoma, and uterine cancers. There was a significant difference in the distribution of CE between the systemic therapy recommendations and the radiation therapy recommendations (χ^2^ statistic 64.16, *P* < .001). Overall, there was a significantly higher proportion of category I CE in the systemic therapy recommendations compared with the radiation therapy recommendations (12.3% vs 9.7%, *P* = .043).

**Conclusions:**

Only 9.7% of radiation therapy recommendations in NCCN guidelines are category I CE. The highest levels of evidence for radiation therapy are in breast and cervical cancers. Despite major advances in the field, these data underline that the majority of NCCN radiation therapy recommendations are based on uniform expert opinion and not on higher level evidence.

## Introduction

Clinical practice guidelines are a crucial tool for providing quality cancer care.^1^ The National Comprehensive Cancer Network (NCCN) clinical guidelines influence medical practice, payor coverage, and standards of care.^2^, ^3^, ^4^, ^5^ Poonacha and Go^6^ analyzed the 2010 NCCN clinical guidelines for the 10 most prevalent cancers in the United States and found that only 6% of all treatment recommendations were category I. A recent update by the same group showed this percentage had increased to just 7% in 2019.^7^ A separate analysis of the NCCN recommendations for anticancer drugs with new Food and Drug Administration approval found 39% of recommendations were not Food and Drug Administration indications.^8^

Radiation therapy is a mainstay treatment modality for many cancers, and it is estimated that 31% of all patients with cancer receive radiation therapy in their first course of treatment and more than 50% require radiation treatment during the management of their disease.^9^^,^^10^ To our knowledge, no prior study has sought to describe the consensus and levels of evidence (CE) concerning radiation therapy recommendations in the NCCN guidelines. The aim of the current work was to assess the levels of evidence underlying the radiation therapy recommendations within the NCCN guidelines. Furthermore, we compared the levels of evidence underlying radiation therapy recommendations with those of systemic therapy.

## Methods and Materials

We identified the 20 most prevalent primary cancer disease sites in the United States with at least 10 radiation therapy specific recommendations in the NCCN guidelines.^11^ We excluded the following disease sites with less than 10 radiation therapy recommendations: basal cell carcinoma, colon cancer, kidney cancer, multiple myeloma, gastric cancer, ovarian cancer, acute myeloid leukemia, chronic lymphocytic leukemia, small bowel adenocarcinoma, testicular cancer, and anal carcinoma. The primary cancer disease sites analyzed were breast, small cell lung cancer, non-small cell lung cancer, prostate, melanoma, bladder, B-cell lymphoma, uterine, pancreas, head and neck, hepatobiliary, thyroid, rectum, central nervous system, esophagus, cervix, soft tissue sarcoma, Hodgkin lymphoma, vulva, and bone.

On August 1, 2020, 2 investigators (M.N., B.J.R.) assessed the NCCN Radiation Therapy Compendium^12^ and obtained the following NCCN guideline versions for each disease site: non-small cell lung cancer was 08/2020; breast, bladder, and rectum were 06/2020; hepatobiliary and esophagus were 05/2020; small cell lung cancer, B-cell lymphoma, and central nervous system (CNS) were 04/2020; prostate, melanoma, thyroid, and vulva were 03/2020; uterine, head and neck, cervix, soft tissue, and Hodgkin lymphoma were 02/2020; and pancreas and bone were 01/2020.

The NCCN guidelines divides its recommendations into 4 CE categories as follows: category I is based on high-level evidence with uniform NCCN consensus, category IIA is based on lower-level evidence with uniform NCCN consensus, category IIB is based on lower-level evidence with NCCN consensus, and category III is based upon any level of evidence with major NCCN disagreement about the recommendation.^13^

We manually recorded the NCCN-defined CE for each recommendation in the aforementioned disease sites (ie, I, IIA, IIB, III). The recommendations were grouped by treatment modality and by treatment purpose as defined by the NCCN (adjuvant, definitive, neoadjuvant, palliative, and consolidative). The NCCN-defined treatment modalities include external beam radiation therapy (EBRT), intensity modulated radiation therapy/volumetric modulated arc therapy, electrons, brachytherapy, accelerated partial breast irradiation, stereotactic body radiation therapy, stereotactic radiosurgery, proton beam radiation therapy, radium-223, Sm-153/Sr-189, intraoperative radiation therapy, and yttrium-90. For data reporting, we grouped intensity modulated radiation therapy, proton beam radiation therapy, and accelerated partial breast irradiation with EBRT. Stereotactic body radiation therapy and stereotactic radiosurgery were grouped as stereotactic treatment. Radioactive nucleotides included radium-223, Sm-153/Sr-189, yttrium-90, and radioactive iodine. We included intraoperative radiation therapy with brachytherapy.

To compare the CE behind NCCN's radiation therapy and systemic therapy guidelines we retrieved data from the NCCN Drugs and Biologics Compendium.^14^ The NCCN Drugs and Biologics Compendium was introduced in 2004 and provides recommendations for the use of oncologic drugs and biological agents in patients with cancer.^15^ As in the NCCN Radiation Therapy Compendium, the NCCN Drugs and Biologics Compendium recommendations are grouped by disease site and offer CE data. We accessed the NCCN Drugs and Biologics Compendium on August 14, 2020. For disease sites with multiple subsites (e.g., head and neck cancer), all subsites were combined. The aforementioned version of the NCCN guidelines was used for both radiation therapy recommendations as well as systemic therapy recommendations to ensure comparative accuracy.

### Statistical methods

We compared the overall distribution of CE between the 2020 NCCN Radiation Therapy Compendium and the 2020 NCCN Drugs and Biologics Compendium using a χ^2^ test. The category I CE were compared from the Radiation Therapy Compendium versus the Drugs and Biologics Compendium across the entire compendia and for each of the 20 disease sites. A two proportion z-test was used to compare the proportion of category I CE between the 2 compendia overall and for each disease site. For the z-test a 2-tailed *P* value was reported. Findings were considered statistically significant if the *P* value was ≤ .05.

## Results

**Fig. 1:**
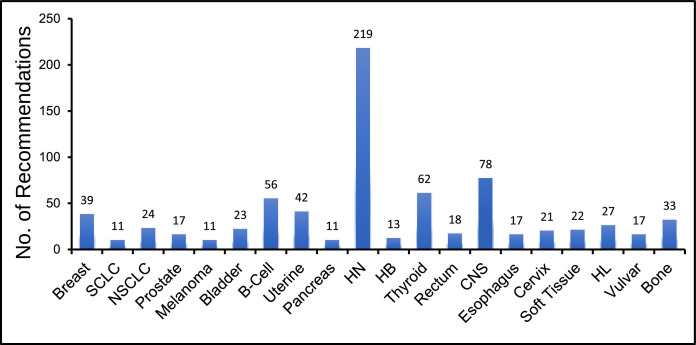
Number of National Comprehensive Cancer Network (NCCN) recommendations for radiation therapy by disease site. Abbreviations: SCLC = Small Cell Lung Cancer; NSCLC = Non-small Cell Lung Cancer; HN = Head and Neck; HB = Hepatobiliary; CNS = Central Nervous System; HL = Hodgkin Lymphoma.

**Fig. 2:**
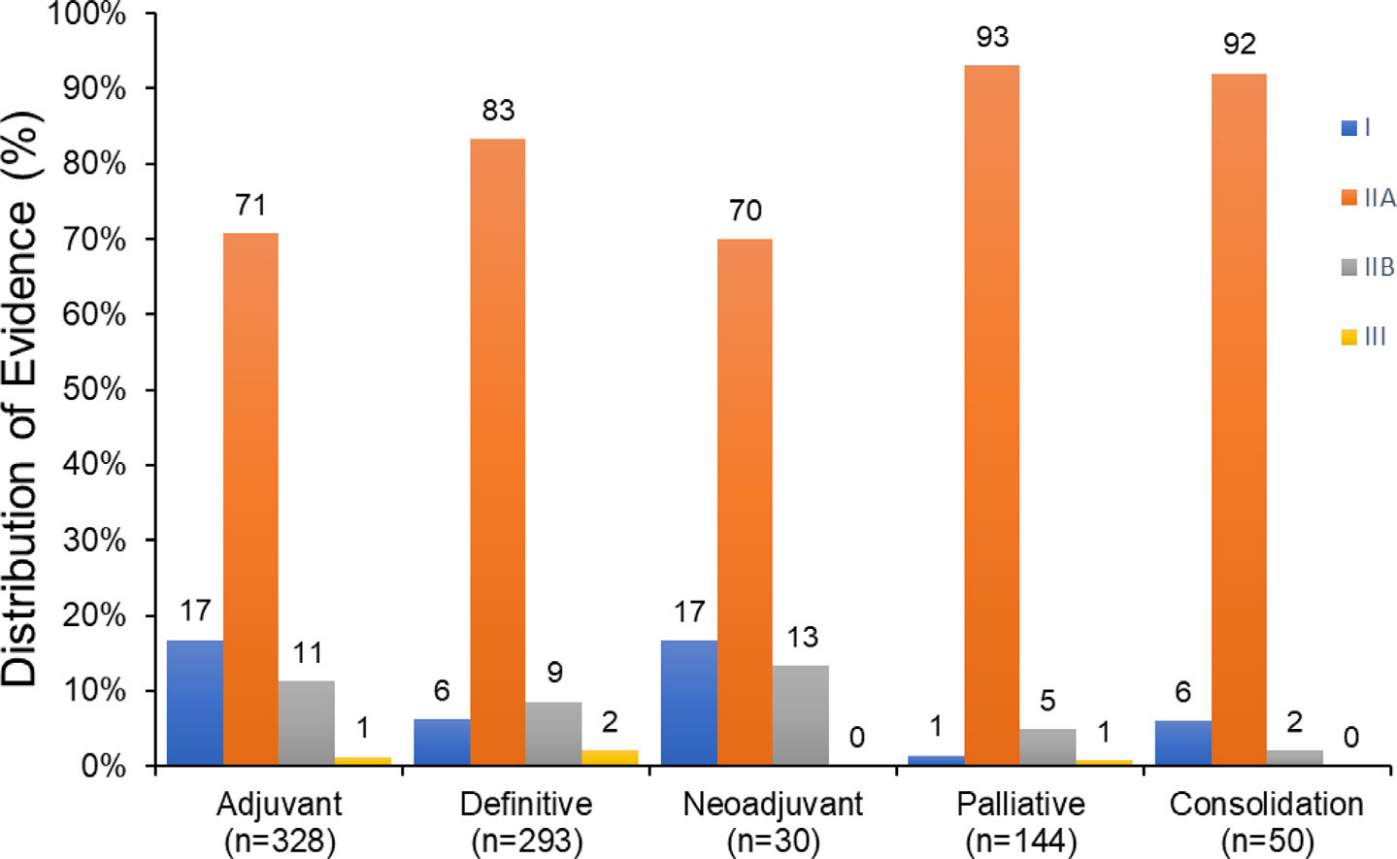
Distribution of National Comprehensive Cancer Network (NCCN) categories of consensus and evidence for radiation therapy treatment purpose; number of recommendations per group is shown (n = number of recommendations in each group).

**Fig. 3:**
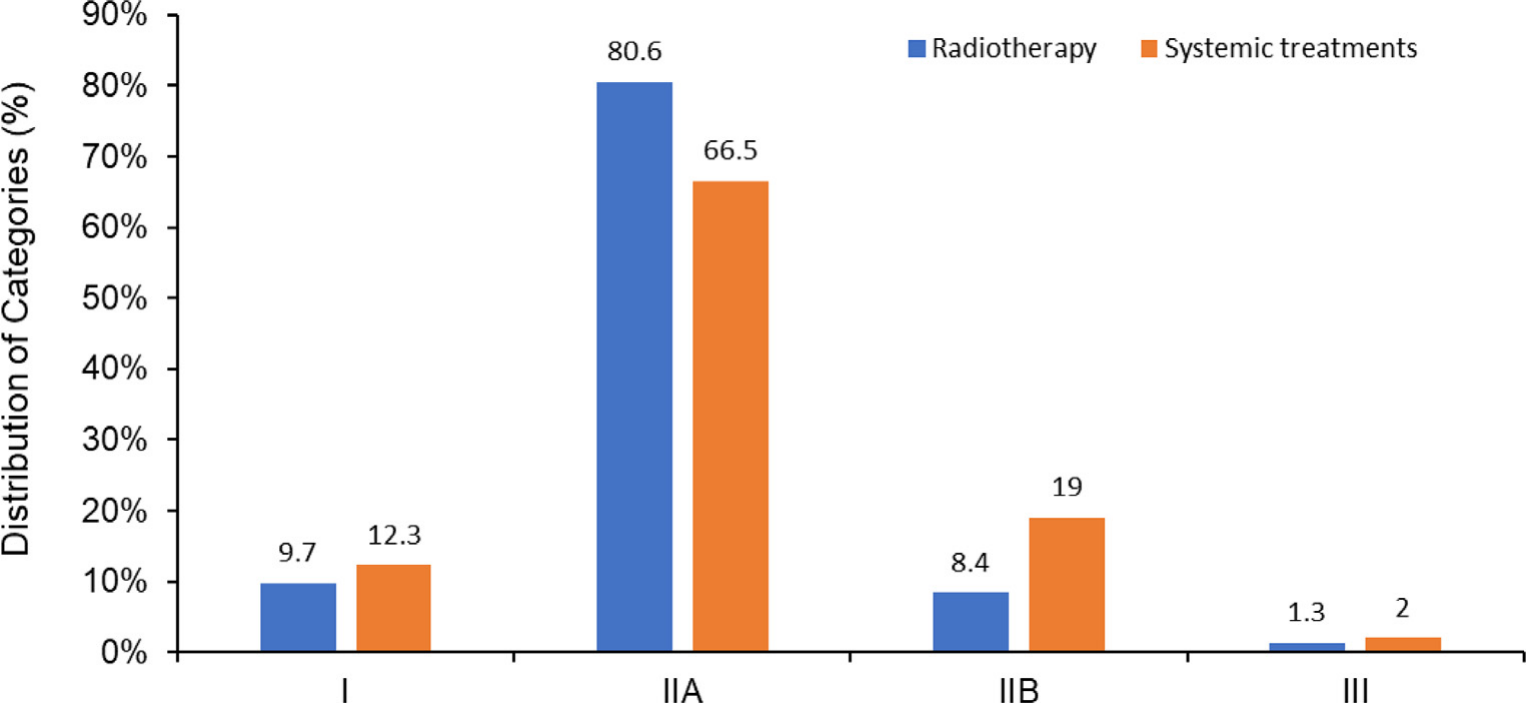
Comparison of National Comprehensive Cancer Network (NCCN) categories of consensus and evidence for radiation therapy versus systemic treatments for all guidelines in 2020.

**Fig. 4:**
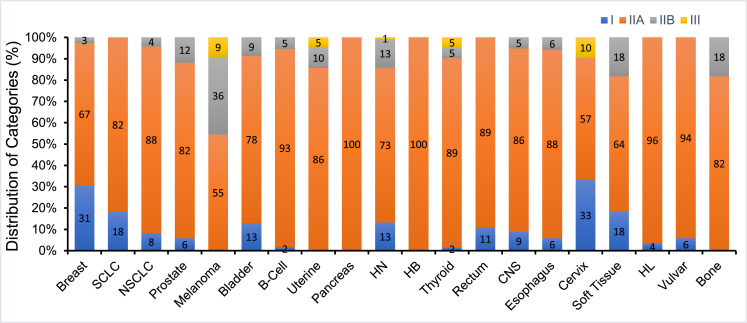
Distribution of National Comprehensive Cancer Network (NCCN) categories of consensus and evidence for radiation therapy according to disease site. Abbreviations: SCLC = Small Cell Lung Cancer; NSCLC = Non-small Cell Lung Cancer; HN = Head and Neck; HB = Hepatobiliary; CNS = Central Nervous System; HL = Hodgkin Lymphoma.

In the 2020 NCCN consensus guidelines there were a total of 761 radiation therapy recommendations from the 20 disease sites reviewed (Fig 1). The disease sites reviewed had a median of 22.5 recommendations (range, 11-219). Most of the recommendations were categorized as definitive or adjuvant with distributions of 39% and 43%, respectively (Fig 2). Among all radiation therapy recommendations for the 20 disease sites, the proportions of category I, IIA, IIB, and III CE were 9.7%, 80.5%, 8.4%, and 1.3%, respectively (Fig 3). The distribution of CE according to recommendations by purpose is shown in Figure 2. Category I recommendations made up only 1% of palliative treatment recommendations. When analyzed by disease site, cervix and breast cancer had the highest proportion of category I radiation therapy recommendations (33% and 31%, respectively). Hepatobiliary, bone, pancreatic, melanoma, and uterine cancers did not have category I CE (Fig 4).

Figures E1a-e and E2 display the CE category distribution according to the recommended NCCN-defined purpose and radiation treatment modality among all guidelines for each disease site. Nearly all definitive and palliative treatment recommendations were based on category IIA CE, except for definitive treatment in melanoma (40% IIA, 60% IIB). CNS (3%) and prostate (33%) were the only disease sites to include category I CE for palliative treatment recommendations. Most of the recommendations were in regard to EBRT (90%) or stereotactic treatment (16%), with only 12% concerning brachytherapy and 5% radioactive nucleotides. All radiation treatment modalities included category I CE: brachytherapy had the highest proportion of category I CE (12%), while EBRT had 10%, radioactive nucleotides 5%, and stereotactic treatment had only 1% category I CE.

**Fig. 5:**
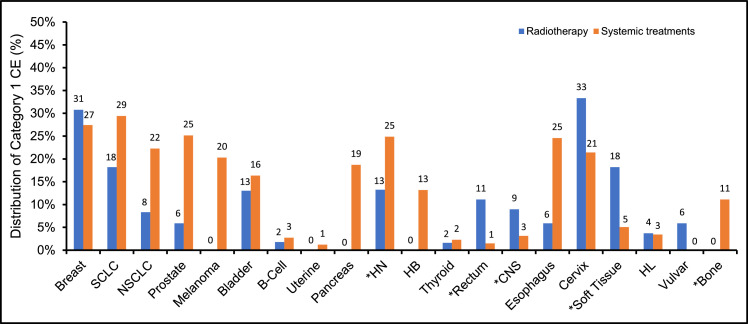
Comparison of National Comprehensive Cancer Network (NCCN) category I consensus and evidence (CE) for radiation therapy versus systemic treatments by disease site. Asterisk denotes disease site with statistically significant difference in the proportion of category I CE between radiation therapy and systemic therapy recommendations using a two proportion, 2-tailed z-test. *P* value < .05 was used for significance. Abbreviations: SCLC = Small Cell Lung Cancer; NSCLC = Non-small Cell Lung Cancer; HN = Head and Neck; HB = Hepatobiliary; CNS = Central Nervous System; HL = Hodgkin Lymphoma.

The distribution of CE in the disease sites for category I, IIA, IIB, and III CE for systemic therapy recommendations was 12.3%, 66.5%, 19.0%, and 1.9%, respectively (Fig 3). There was a significant difference in distribution of the category of CE between the 2020 NCCN radiation therapy versus Drugs and Biologics recommendations (χ^2^ statistic 64.16, *P* < .001). There was a significantly higher proportion of category I CE in the systemic therapy recommendations compared with the radiation therapy recommendations (12.3% vs 9.7%, *P* = .043). In 5 of the 20 analyzed disease sites there was a significant difference in category I CE radiation therapy versus systemic therapy recommendations (Fig 5): head and neck (13% vs 25%, *P* < .001), rectum (11% vs 1%, *P* = .016), CNS (9% vs 3%, *P* = .039), soft tissue (18% vs 5%, *P* = .017), and bone (0% vs 11%, *P* = .046).

## Discussion

Prior studies of the NCCN guidelines found most of the recommendations are based on lower levels of evidence, though none of those studies focused on radiation therapy.^6^^,^^7^ In this analysis of the NCCN guidelines specific to radiation therapy, we found that only 9.7% of current recommendations are category I CE. There is a large amount of variation between disease sites. For instance, breast and cervical cancers have the highest levels of category I CE radiation therapy recommendations. In contrast, hepatobiliary, bone, pancreatic, melanoma, and uterine cancers all lack category I CE recommendations. In comparison to systemic therapy, bone and head and neck cancers had significantly fewer category I CE radiation therapy recommendations, while other disease sites such as the rectum, CNS, and soft tissue had significantly more category I CE radiation therapy recommendations. Taken together, these findings highlight disease sites where resources are necessary to produce higher levels of evidence specific to radiation therapy through higher quality randomized controlled trials.

The majority of radiation therapy guidelines were category IIA (81%), with just 8% and 1% as category IIB and III, respectively. The category I evidence ranged from 17% with adjuvant and neoadjuvant intent to 1% in the palliative setting. In regards to radiation treatment modality, the distribution of category I CE evidence ranged from 12% with brachytherapy recommendations to just 1% category I CE with stereotactic radiation therapy. These findings further underscore areas within radiation oncology where higher quality levels of evidence are needed. In the 20 disease sites evaluated, there were large disparities in category I evidence. For example, cervical and breast cancer had 33% and 31% category I radiation therapy recommendations, respectively, while hepatobiliary and bone cancers had no category I radiation therapy recommendations.

When the NCCN Radiation Therapy Compendium and Drugs and Biologics Compendium were compared, there was a statistically significant difference between their categories of evidence and consensus. There was a significantly higher proportion of category I CE in the Drugs and Biologics recommendations compared with the NCCN Radiation Therapy recommendations. Furthermore, 5 of the 20 analyzed disease sites had significant differences in the proportion of category I CE recommendations. The head and neck and bone had significantly fewer category I radiation therapy recommendations, while the CNS, rectum, and soft tissue had significantly fewer category I systemic recommendations. It should be noted that this comparison speaks to the proportional quality but not absolute quantity of recommendations.

The NCCN clinical guidelines are pervasive in guiding oncologic care in the United States and internationally. Adherence to the NCCN guidelines is associated with improved patient outcomes and has become an indicator of quality of care.^4^^,^^16^, ^17^, ^18^ Nonetheless, previous analyses of the entire NCCN clinical practice guidelines found that just 6% of recommendations were category I in 2010 and 7% in 2019.^6^^,^^7^ The NCCN Radiation Therapy Compendium, albeit with different sites of disease, compares favorably with 9.7% category I CE. However, the NCCN Drugs and Biologics Compendium had 12.3% category I CE.

The reliance on new technologies within radiation oncology poses unique challenges in generating level I evidence based on randomized controlled data.^19^^,^^20^ Once technology becomes available, the community tends to quickly adopt certain practices based on technological benefits before proof of patient benefit is derived from higher level studies. In addition, the field of radiation oncology is grossly underfunded in the United States relative to its role in cancer care. The proportion of National Institutes of Health funding that goes into radiation research is only 1.6% of the National Institutes of Health budget provided for cancer research.^21^ Given these headwinds, it is perhaps not surprising that the Drugs and Biologics Compendium has a greater reliance on category I recommendations. Systemic therapy trials are heavily supported by industry funding, and previous work has addressed whether financial conflicts of interest have affected NCCN recommendations.^22^^,^^23^

This analysis, nonetheless, highlights the need for more investment in high quality randomized trials in radiation oncology to address the clinical benefit and also the added benefit of alternative technologies (ie, proton therapy vs photons). Well-conducted randomized controlled trials are the only method to reliably test new treatment interventions, and this painstaking process should not be replaced by other strategies like population-based observational studies.^24^ Based on the present study, stereotactic radiation therapy and radiosurgery are modalities with the greatest need for level 1 evidence. Indeed, several large phase III randomized trials are currently investigating these modalities.^25^, ^26^, ^27^, ^28^

Private insurers and the Centers for Medicare and Medicaid Services rely on the NCCN clinical practice guidelines for reimbursement policies.^2^ Because only 9.7% of the NCCN Radiation Therapy Compendium recommendations analyzed in this study are category I CE, overreliance on the clinical practice guidelines may stifle appropriate clinical care.

This study has several limitations. First, our analysis relies on the NCCN-derived categories to determine the category of CE. NCCN does not offer granularity on how high- or low-level evidence is defined and how consensus is made. Per the NCCN website, “The development of the NCCN Guidelines is an ongoing and iterative process, which is based on a critical review of the best available evidence and derivation of recommendations by a multidisciplinary panel of experts in the field of cancer.” NCCN posts short summaries of the meetings and panel discussions as well as disclosures of conflicts of interest. Second, our data are limited to the 20 most prevalent disease sites with at least 10 recommendations in the Radiation Therapy Compendium and may not be reflective of the entire compendium. Limiting the disease sites to those with at least 10 radiation therapy recommendations may have introduced bias into the comparison between the Radiation Therapy Compendium and Drugs and Biologics Compendium. In some of the disease sites without 10 radiation therapy recommendations, for instance, radiation therapy is an important curative-intent modality (e.g., anal carcinoma, basal cell carcinoma).

## Conclusions

In conclusion, this analysis found that only 9.7% of the radiation therapy recommendations in the 2020 NCCN guidelines are category I CE, with most of the recommendations (81%) category IIA. The distribution of categories of evidence and consensus recommendations was significantly different for radiation therapy and systemic treatments, with the latter having more category I recommendations. Despite major advances in the field of radiation therapy, these data underline that the majority of NCCN recommendations are based on uniform expert opinion and not on higher level evidence.

## Appendix Supplementary materials

Image, application 1
