# Supplementary material for: Levels of Evidence for Radiation Therapy Recommendations in the National Comprehensive Cancer Network (NCCN) Clinical Guidelines
Source: Adv Radiat Oncol. 2021 Oct 29;7(1):100832. doi: 10.1016/j.adro.2021.100832 (PMC8626664; doi:10.1016/j.adro.2021.100832)
Supplement: Supplementary file 1 [file mmc1.pdf]

Supplementary Figures

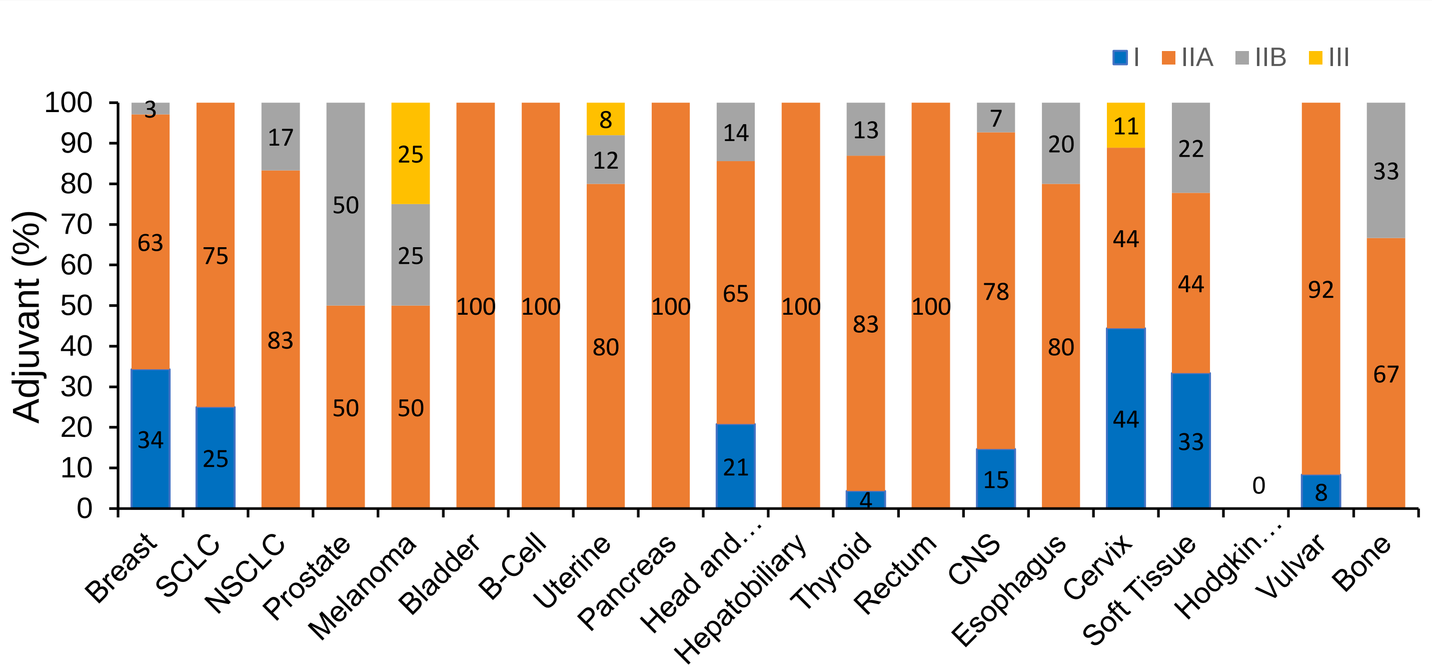

Supplementary Figure 1A. Distribution of NCCN categories of consensus and evidence for adjuvant radiation therapy according to disease site.

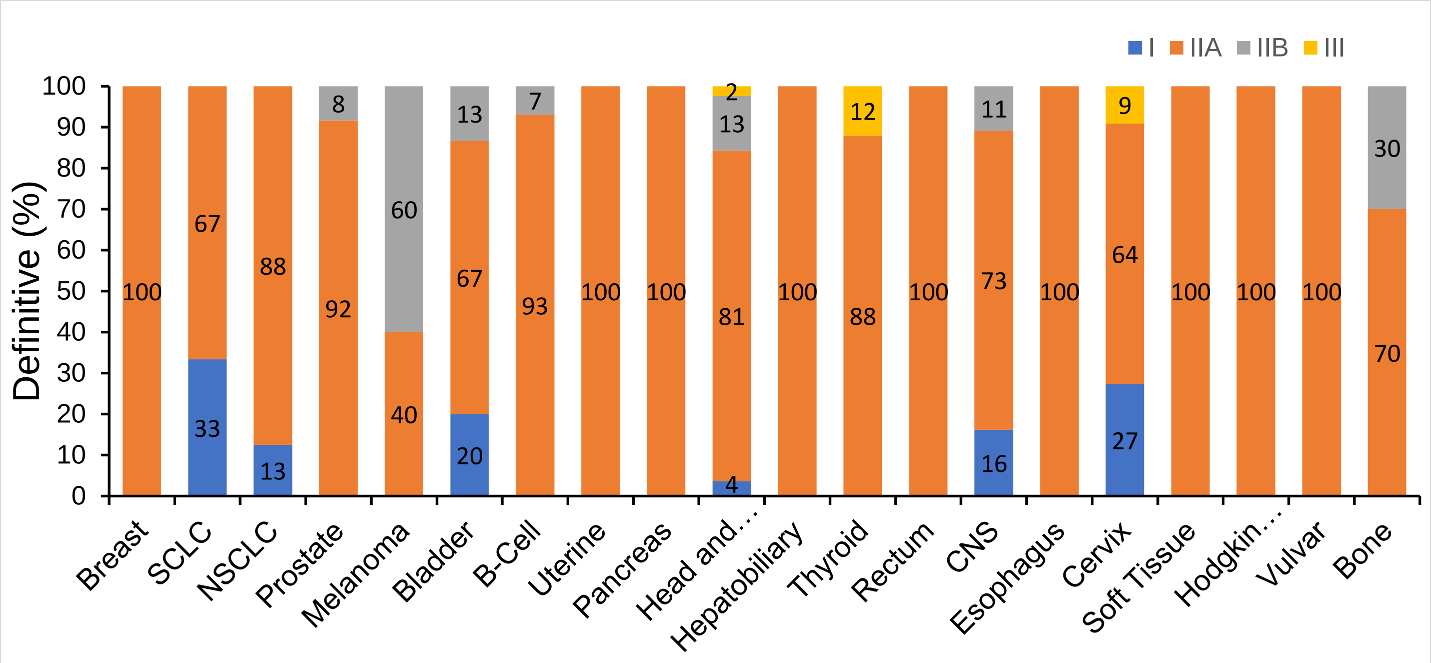

Supplementary Figure 1B. Distribution of NCCN categories of consensus and evidence for definitive radiation therapy according to disease site.

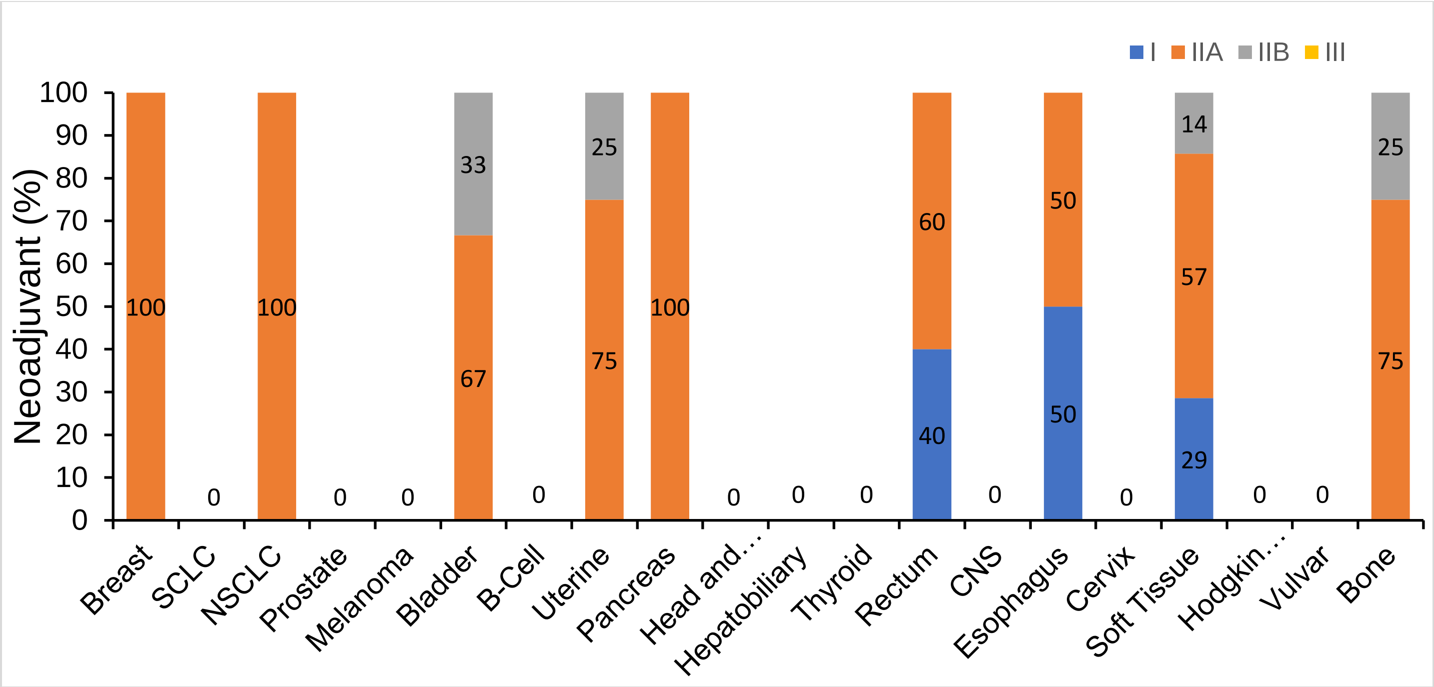

Supplementary Figure 1C. Distribution of NCCN categories of consensus and evidence for neoadjuvant radiation therapy according to disease site.

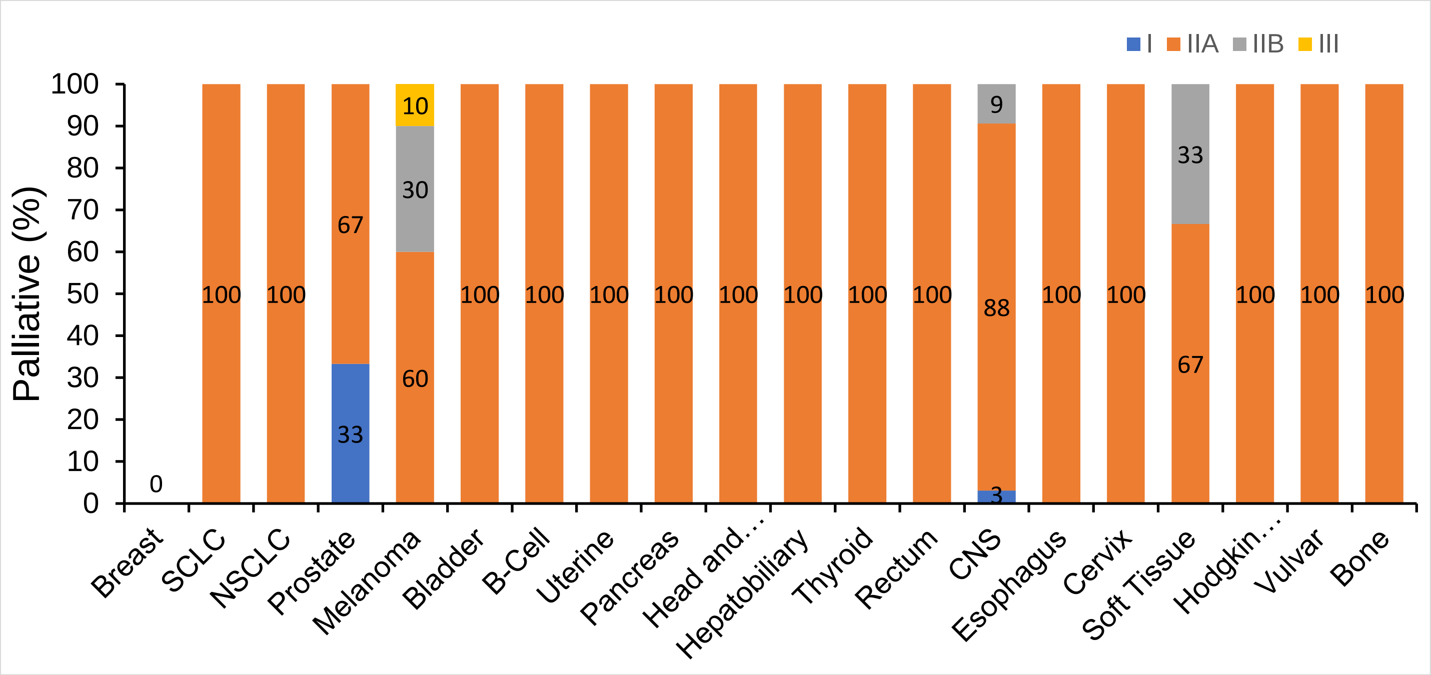

Supplementary Figure 1D. Distribution of NCCN categories of consensus and evidence for palliative radiation therapy according to disease site.

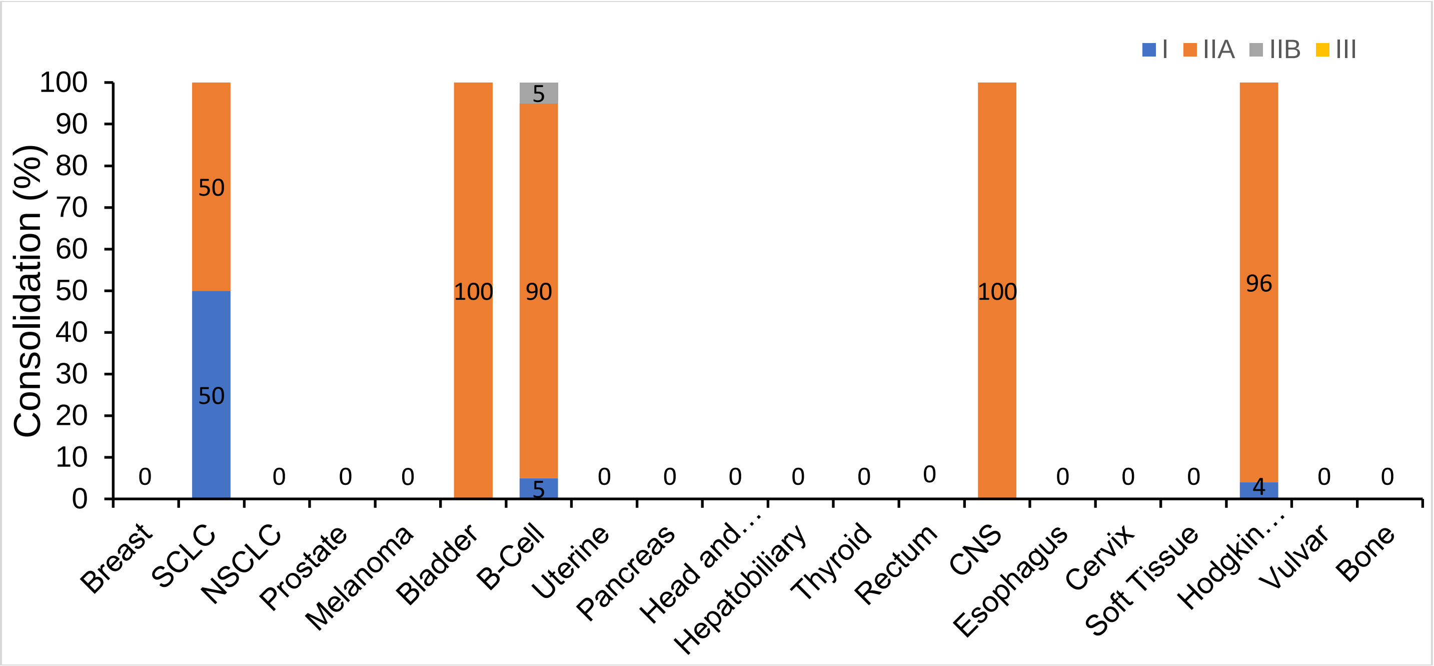

Supplementary Figure 1E. Distribution of NCCN categories of consensus and evidence for consolidative radiation therapy according to disease site.

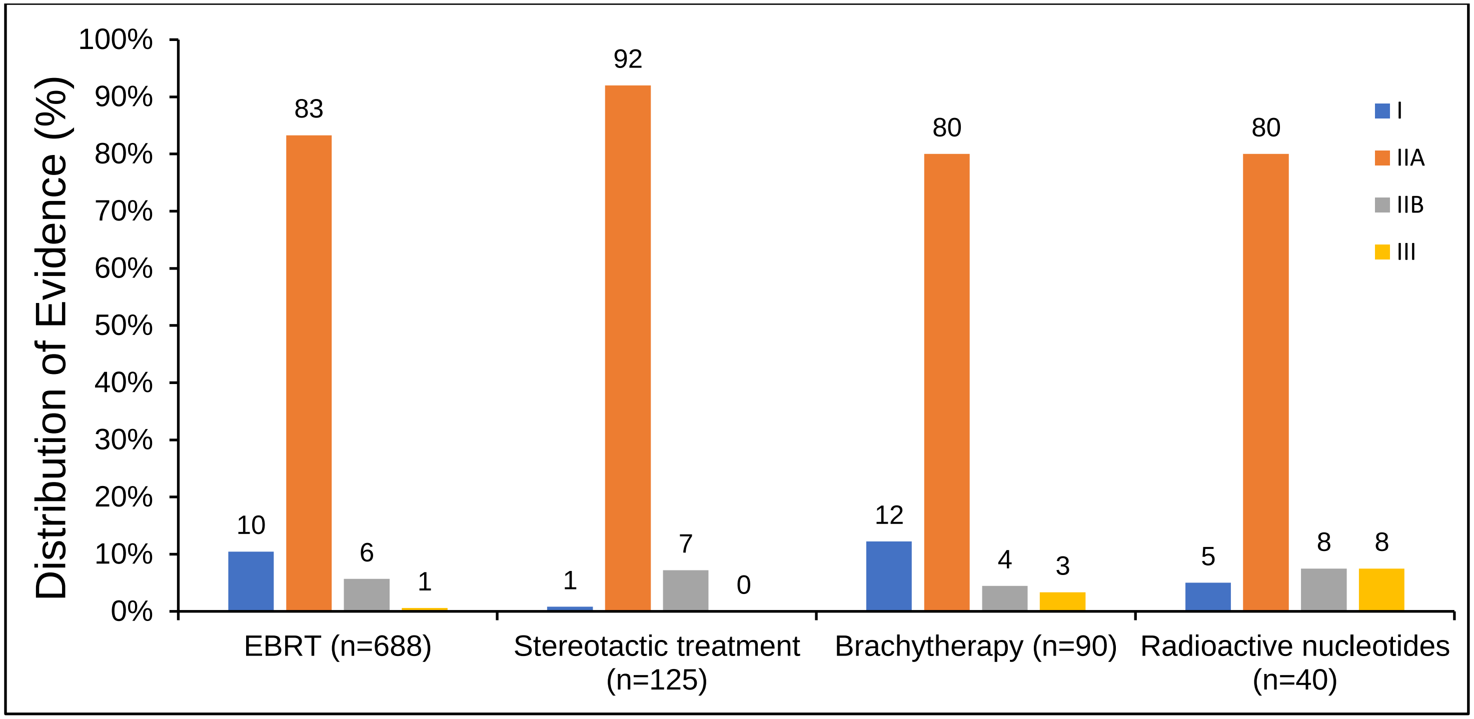

Supplementary Figure 2. Distribution of NCCN categories of consensus and evidence for radiation therapy by treatment modality; number of recommendations per group is shown (n=*).

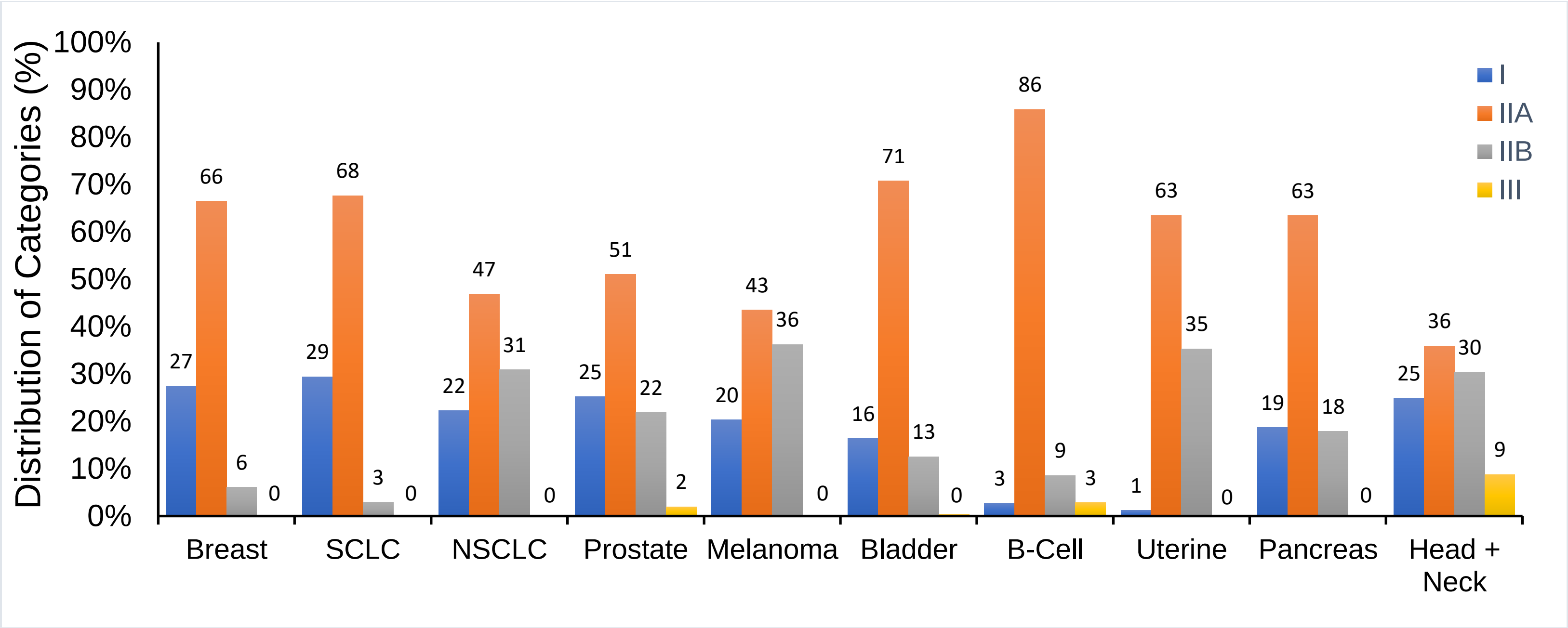

Supplementary Figure 3A. Distribution of NCCN categories of consensus and evidence for systemic treatments according to disease site.

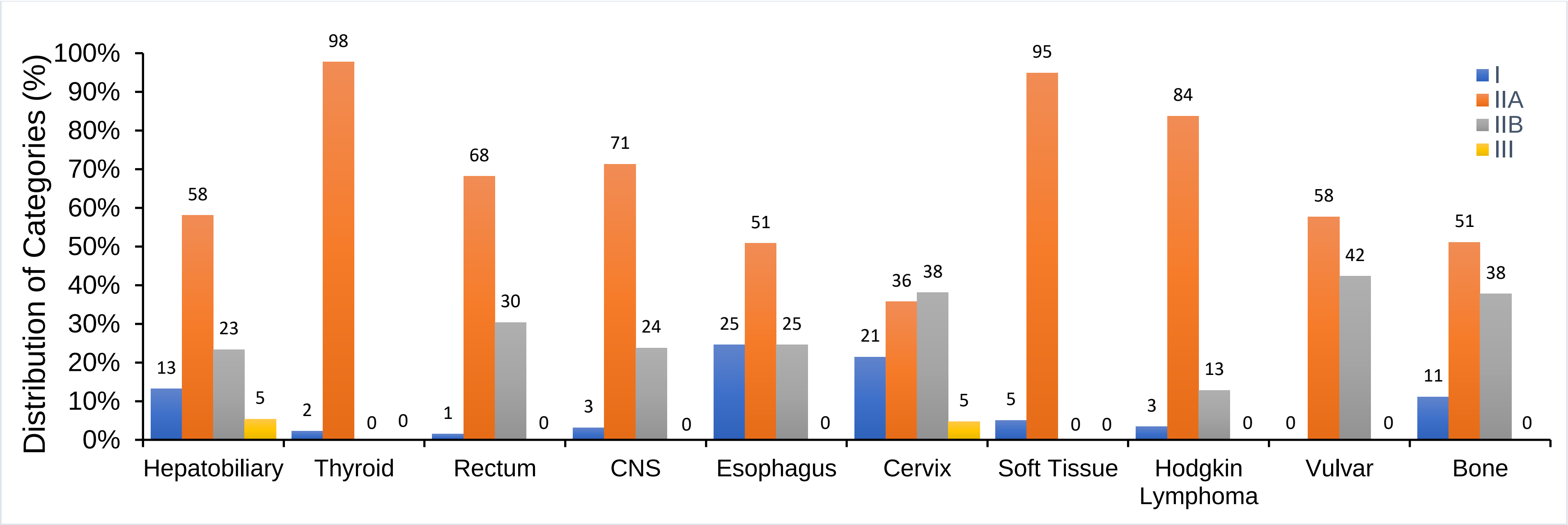

Supplementary Figure 3B. Distribution of NCCN categories of consensus and evidence for systemic treatments according to disease site.

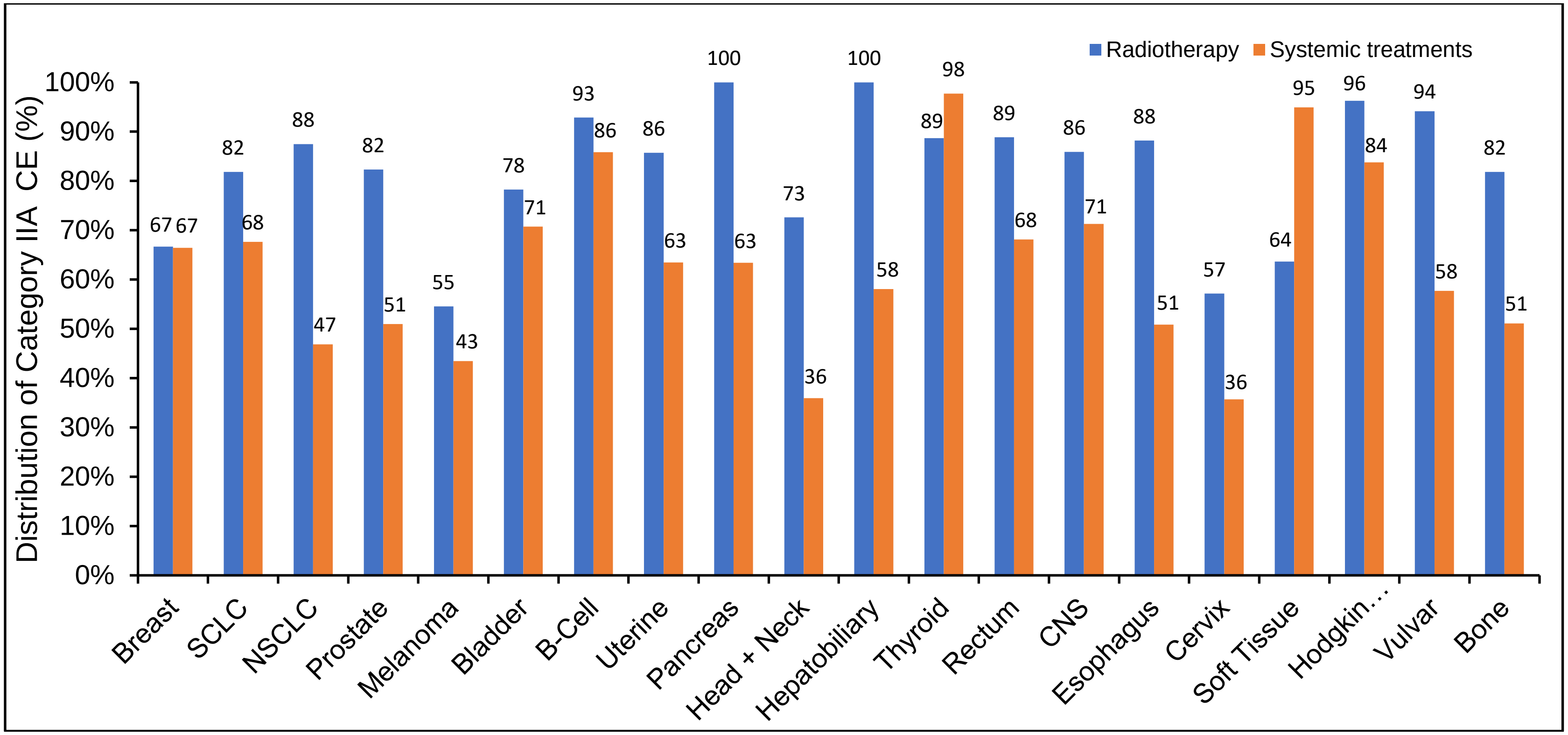

Supplementary Figure 4. Comparison of NCCN Category IIA consensus and evidence for radiation therapy versus Drugs and Biologics Compendium treatment by disease site.

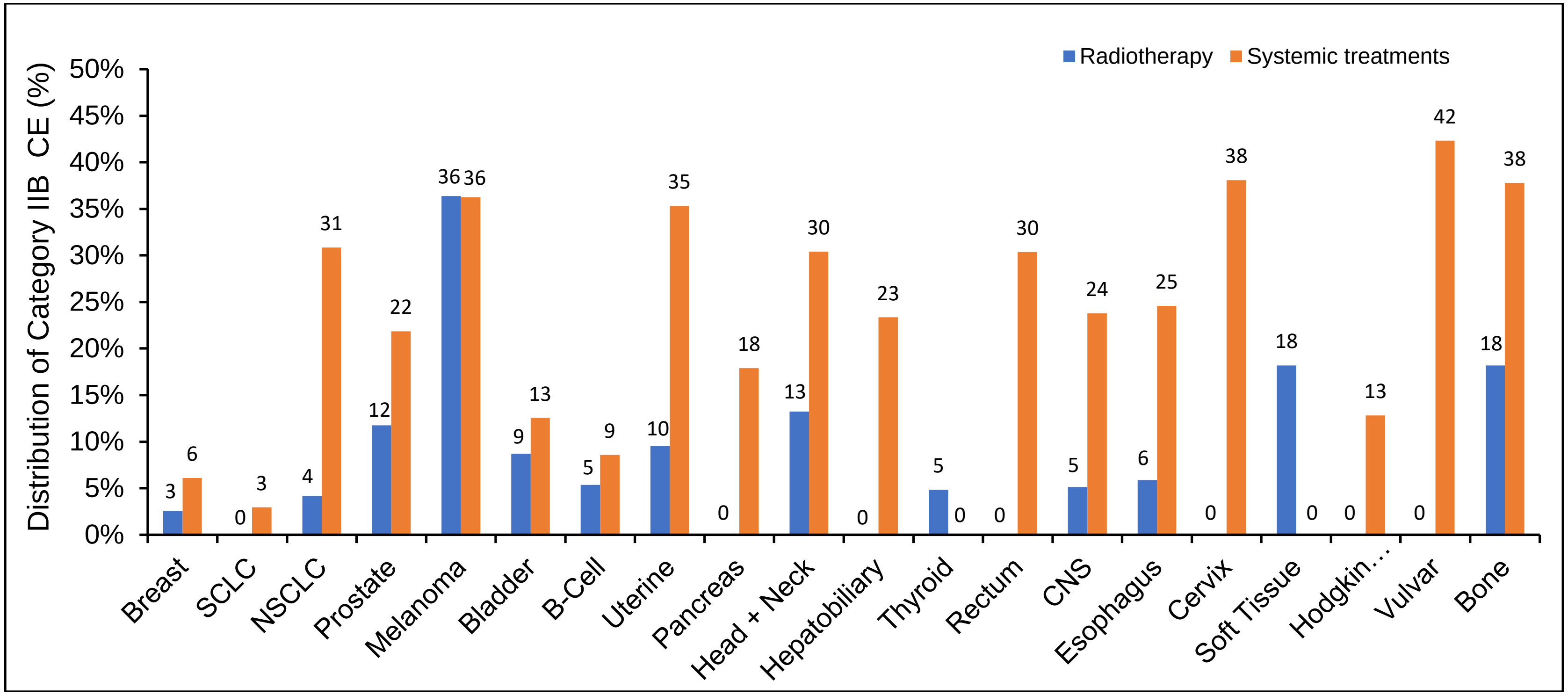

Supplementary Figure 5. Comparison of NCCN Category IIB consensus and evidence for radiation therapy versus Drugs and Biologics Compendium treatment by disease site.

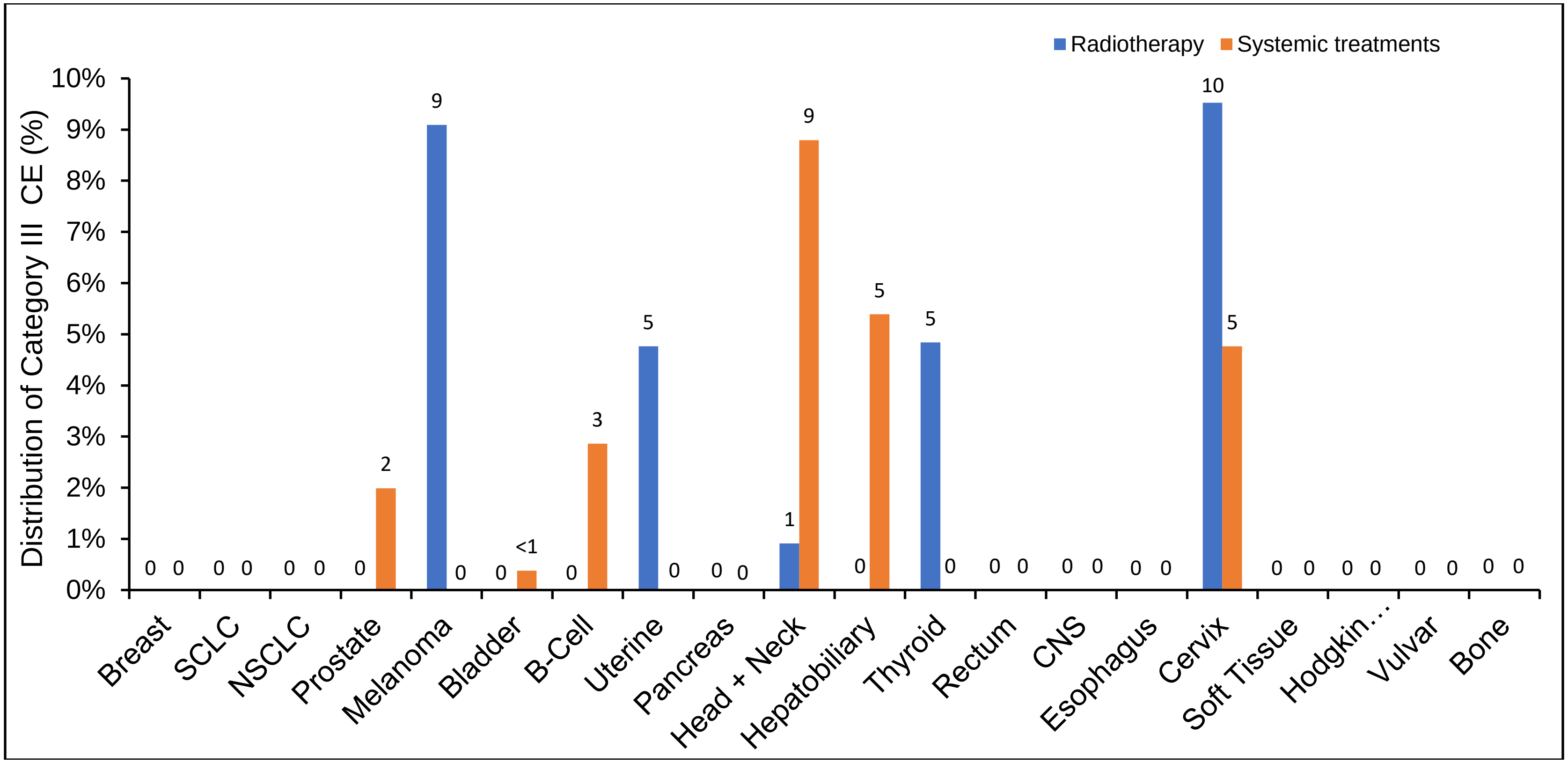

Supplementary Figure 6. Comparison of NCCN Category III consensus and evidence for radiation therapy versus Drugs and Biologics Compendium treatment by disease site.
